# Integrated Proteome and Cytokine Profiles Reveal Ceruloplasmin Eliciting Liver Allograft Tolerance via Antioxidant Cascades

**DOI:** 10.3389/fimmu.2018.02216

**Published:** 2018-09-26

**Authors:** Pei-Wen Wang, Tung-Ho Wu, Tai-Long Pan, Mu-Hong Chen, Shigeru Goto, Chao-Long Chen

**Affiliations:** ^1^Department of Medical Research, China Medical University Hospital, China Medical University, Taichung, Taiwan; ^2^Division of Cardiovascular Surgery, Veterans General Hospital, Kaohsiung, Taiwan; ^3^School of Traditional Chinese Medicine, Chang Gung University, Taoyuan, Taiwan; ^4^Chinese Herbal Medicine Research Team, Healthy Aging Research Center, Chang Gung University, Taoyuan, Taiwan; ^5^Liver Research Center, Chang Gung Memorial Hospital, Taoyuan, Taiwan; ^6^Research Center for Food and Cosmetic Safety and Research Center for Chinese Herbal Medicine, Chang Gung University of Science and Technology, Taoyuan, Taiwan; ^7^Department of Psychiatry, Taipei Veterans General Hospital, Taipei, Taiwan; ^8^Department of Psychiatry, College of Medicine, National Yang-Ming University, Taipei, Taiwan; ^9^Department of Surgery, Kaohsiung Chang Gung Memorial Hospital, Kaohsiung, Taiwan

**Keywords:** ceruloplasmin, oxidant-antioxidant, liver transplantation, apoptosis, 2-de oxyblot

## Abstract

Acute rejection (AR) and spontaneous tolerance may occur after allograft orthotopic liver transplants (OLT) performed in certain combinations of donor and recipient rat strains, yet the underlying molecular cascades involved in these conditions remain poorly understood. Comprehensive analysis with proteomic tools revealed that ceruloplasmin was highly expressed during the tolerant period on day 63 post-OLT (POD 63) compared to the rejected samples on POD 14. Meanwhile, cytokine expression profiles implied that the inflammation was significantly stimulated in the AR subjects. Again, protein carbonylation was dramatically upregulated in the rejected subject within the tolerant group. Knockdown of ceruloplasmin would elicit more severe ROS damage, leading to cell death in the presence of H_2_O_2_, which induced Nrf2 cascade and the recovery of ceruloplasmin to mediate spontaneous tolerance. In summary, ceruloplasmin may contribute to amending the oxidative stress that eventually causes cell apoptosis and to maintaining the survival of hepatocytes in a drug-free tolerance OLT model.

## Introduction

Orthotopic liver transplantation (OLT) has become a definitive therapeutic option in patients with acute or chronic liver disease while immune rejection is still a major cause of operation failure and mortality (1–3). It has been reported that spontaneous tolerance could be induced between different rat strains after liver transplantation (4, 5). Although an early rejection reaction occurs over the first 2 weeks under OLT (DA-PVG), drug-free tolerance would be induced on 63 days (POD 63). Conversely, all recipients die of acute rejection within 14 days as the same DA donor livers are transplanted into LEW recipients. In spite of some strong reasons behind this phenomenon, the exact mechanism of this unique tolerance remains unknown (6–8). Moreover, the underlying cascades and response elements have not yet been fully revealed.

During different stages of liver allograft rejection or tolerance, huge numbers of immune cells, serum proteins, and molecules associated with immune responses will change in quantity and quality (9–12). Thus, high throughput proteomics technology designed to deal with this complexity was used to identify the large-scale molecular pathways and pathological mechanisms involved in the OLT. Previous study also demonstrated that several proteins, including haptoglobin, immunoglobulin, and the specific glycoproteins, should mediate the acquisition of self-tolerance (13, 14). However, other parts of the puzzle having a role in the immune responses should be implied since those proteins could not completely relieve the rejection after OLT.

Numerous documents have implied that inflammatory responses should cause the induction and progression of hepatic injury after OLT (15–17). Many reports have indicated that highly reactive oxygen species (ROS) play a pivotal role in tissue damage of inflammatory organisms. Increased oxidative stress caused by acute inflammation would subsequently result in protein modifications and alteration of the amino acid sequence, leading to protein dysfunction and organ damage (18, 19). Next, impairment of the antioxidant proteins due to oxidative damage would cause the cells or tissues to be more vulnerable in the presence of inflammatory species such as cytokines and free radicals (20–22). Therefore, cytokine arrays were applied to monitor the recruitment of various cytokines/chemokines that reflect specific events in response to severe rejection after OLT. The functional “signature network” using MetaCore™ pathway analysis tools produces overall cellular mechanisms derived from differences in protein and cytokine levels. This method simultaneously delineates the signaling pathways in view of the architecture to represent biological functionality and the integration of molecular and clinical information (23, 24).

In the current study, the comprehensive analysis of global changes in rat serum provides a feasible method for detecting possible mechanisms that play a critical role in providing immunological privilege in the OLT model. Our findings may provide important information for therapeutic designs after transplantation and enhance survival rate by predicting disease progression as well as treatment responses.

## Materials and methods

### Orthotopic liver transplantation

Five DA (RT1^a^), five PVG (RT1^c^) and Five LEW (RT1^l^) inbred strains of male rats, weighing 200-250g, were housed at the SPF animal facility and allowed free access to water and standard rat chow. All the animal experiment and associated protocol were approved in compliance with the standards of Chang Gung University's Committee for the Use and Care of Animals. The Committee recognizes that the proposed animal experiment follows the guideline as shown in the “Guide for Laboratory Animal Facilities and Care” as promulgated by the Council of Agriculture. Orthotopic liver transplantation (OLT) was performed under ether anesthesia using Kamada's method with some modification (11, 25). DA livers were orthotopically transplanted into PVG recipients and the graft would overcome rejection without any immunosuppressive drugs (26). As a syngenic control, five DA donor livers were transplanted into five DA recipients. The blood and liver samples were obtained and stored in −80°C for further analysis.

### TUNEL assay

Apoptosis was assessed by terminal deoxynucleotidyl transferase- mediated dUTP biotin nick end labeling (TUNEL) using ApopTag® Plus Peroxidase *in situ* Apoptosis Detection Kit (Millipore) according to the manufacturer's instructions. The slides was counterstained with eosin and mounted. The numbers of stained and unstained cells were then counted from randomly chosen fields per slide within a high-power field (×200 magnifications) under an Olympus BX50 microscope. Image-Pro plus 4.5 (Media Cybernetics) image analysis software was used to quantify image signals (27).

### Two-dimensional electrophoresis, image, and mass spectrometric analysis

Aliquot serum (150 μg) samples were diluted in IPG sample buffer and rehydrated for 12 hr at 30 V. Isoelectric focusing (IEF) was conducted automatically with a total voltage of 90 kVhr. The second dimensional electrophoresis was carried out on 10 % acrylamide gels (Bio-Rad) at 40 mA. Proteins were visualized by silver staining and quantified using the Prodigy SameSpots software (Nonlinear Dynamics). Spots of interest were excised and in-gel digested with trypsin according to previously described procedures (13, 27). MS analysis was performed on an Ultraflex MALDI-TOF mass spectrometer (Bruker-Daltonik). Monoisotopic peptide masses were assigned and used for database searches with the Mascot search engine (http://www.matrixscience.com). Search parameters were set as follows: a maximum allowed peptide mass error of 50 ppm, and consideration of one incomplete cleavage per peptide (28).

### Rat cytokine protein array

The spectrum of cytokines produced by apparently rejection and tolerance subjects was determined using an antibody-based protein microarray (RayBio Rat Cytokine Ab Array I and 1.1 Map, RayBiotech Inc.,) designed to detect 19 growth factors, cytokines and chemokines. Proteins were detected via an enhanced chemiluminescence procedure according to previously described procedures (29). By subtracting the background staining and normalizing to the positive controls on the same membrane, we obtained the relative protein concentrations.

### Biological network analysis using metacore™

Applied MetaCore™ software (vers. 5.2 build 17389, GeneGo, St. Joseph, MI, USA) was applied to reveal associated ontological classes and relevant pathways which were represented among the proteins identified by the mass fingerprint and cytokine array (28).

### RNA isolation and northern blot

Total RNA was extracted from the rat liver using a TRIzol Reagent, and dissolved in diethylpyrocarbonate (DEPC)-treated water. Equal amount of total RNA was separated on denaturing formaldehyde agarose gels and blotted onto nylon membranes. Hybridization and detection were performed using detection kit (Thermo Fisher Scientific) according to the supplier's instruction. The β-actin transcript was used as an internal control to normalize the concentration of mRNA in each sample (30, 31). Transcript intensities were recorded as digitalized images using a high-resolution scanner and quantity by GeneTools Image Software (Syngene).

### 2-DE oxyblot

Following isoelectric focusing, IPG strips were incubated in solution of 2N HCl with 10 mM 2,4-dinitrophenylhydrazine (DNPH) at 25°C for 20 min. After carbonyl derivatization step, strips were washed with 2 M Tris-base/30% glycerol for 15 min and then used for molecular weight dependent separation of proteins by SDS-PAGE, followed by protein blotting to a membrane. Next, this membrane was incubated overnight at 4°C with the anti-DNP (Sigma) in the TBST containing 5% non-fat milk. The blots were then incubated with goat anti-rabbit IgG/HRP conjugate for 2 hr at room temperature. Enhanced chemiluminescence (PerkinElmer) was used for detection (24, 27).

### Western blot analysis

Protein was separated with 10% SDS-PAGE and transferred to a PVDF membrane which was incubated with anti-ceruloplasmin, anti-PCNA, anti-caspase-3, anti-SOD-1, anti-catalase, anti-Nrf2, anti-HO-1 and anti-β-actin (Santa Cruz) overnight at 4°C. Blots were washed and incubated with HRP-labeled secondary antibody. After washing in TBST, enhanced chemiluminescence was used for protein detection. The band intensity was quantified using GeneTools Image Software (Syngene, UK), and β-actin was used as an internal control (32). The Western blot experiments were repeated in triplicate.

### Effects of ceruloplasmin silencing on cell survival under H_2_O_2_ treatment

HepG2 cells were plated onto 24-well plates (2 × 10^4^ cells/well), maintained in antibiotic-free medium for 6 hr, and transfected with a mixture containing Opti-MEM, 8 μL/well Lipofectamine 2000 (Invitrogen, San Diego, CA) and 0.5 μg/well a mixture of three ceruloplasmi siRNA. At 24 hr post-transfection, cells were pretreated with either 1 or 5 mM H_2_O_2_ dissolved in medium for another 24 hr. Then, the cells were harvested for MTT assays and Western blot analyses (27, 32). Results shown are the representative of three separate experiments.

### Statistical analysis

All values were presented as the mean ± standard deviation (SD). Statistical analysis of the mean values was carried out with the ANOVA using SPSS software (SPSS Inc., USA). Differences were considered as significant at ^*^p < 0.05.

## Results

### Validating the hepatic apoptosis via TUNEL histopathology

**Figure 1:**
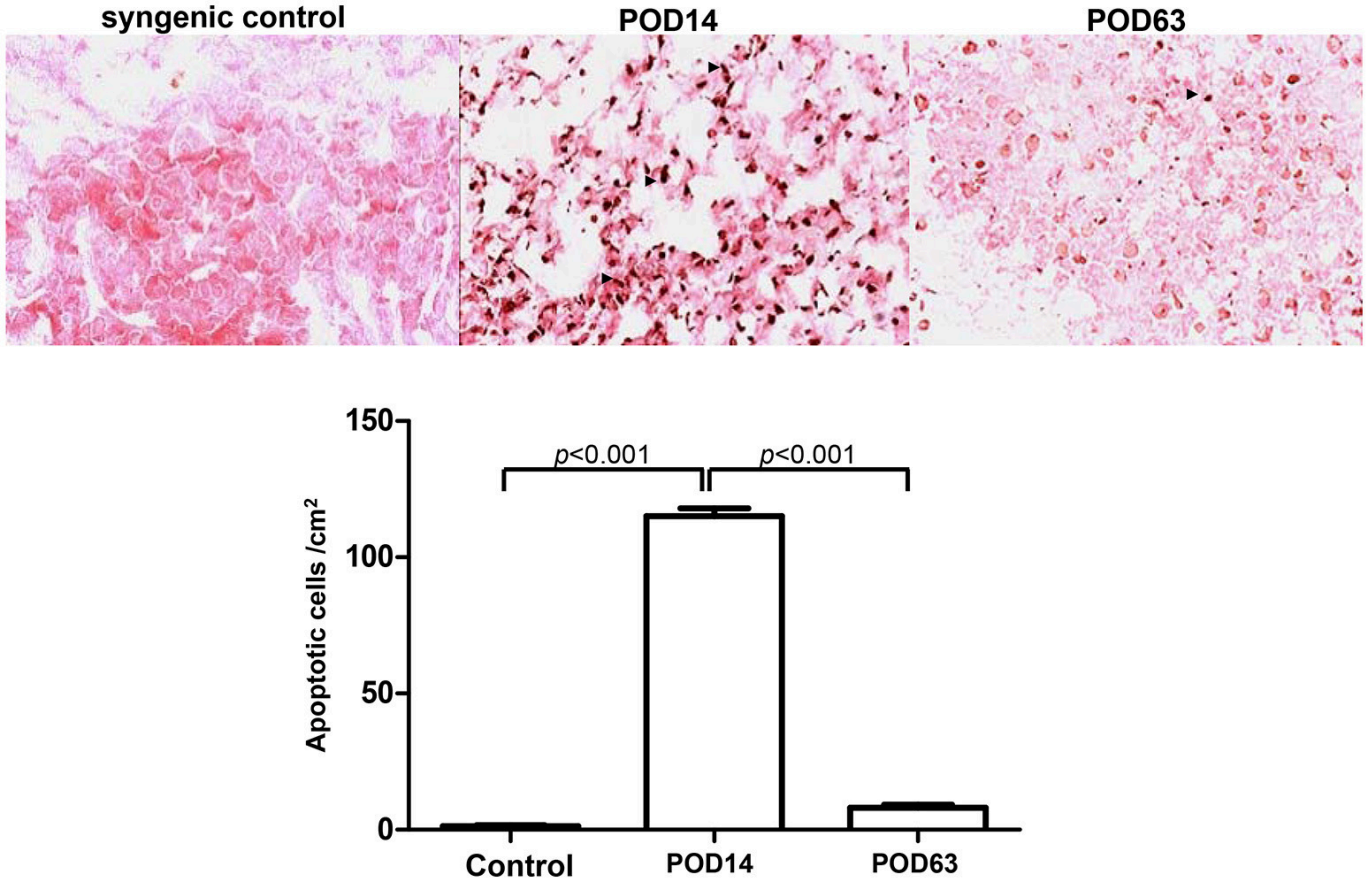
Histological analysis of cellular apoptosis in rat liver tissues on syngenic control, POD14 and POD63. The apoptotic signal was quantified and indicated by the bar chart.

Histological investigation was performed to reveal *in situ* apoptotic conditions in the allogeneic transplant group. Based on previous findings, it was thought that severe AR, demonstrated by lymphocyte and eosinophils infiltration into the liver parenchyma around the portal fields and a great number of foci of necrotic hepatocytes, would occur within the liver lobules at POD14. On the contrary, sinusoidal lymphocytes had disappeared and the liver tissue was restored to a normal histological architecture with no signs of inflammation at POD63, indicating that rejection had been spontaneously overcome. Moreover, intact lobular architecture and hepatic cells were observed in the syngenic control. As expected, the terminal deoxynucleotidyl transferase dUTP nick-end labeling (TUNEL) positive apoptotic signal was dramatically induced in the rejection samples (POD14). Conversely, no significant increase in apoptotic cells was observed in the tolerant subjects (POD63) and rare signal was detected in the control samples (Figure 1).

### Differential display of serum protein profiles by analytical two-dimensional electrophoresis (2-DE)

**Figure 2:**
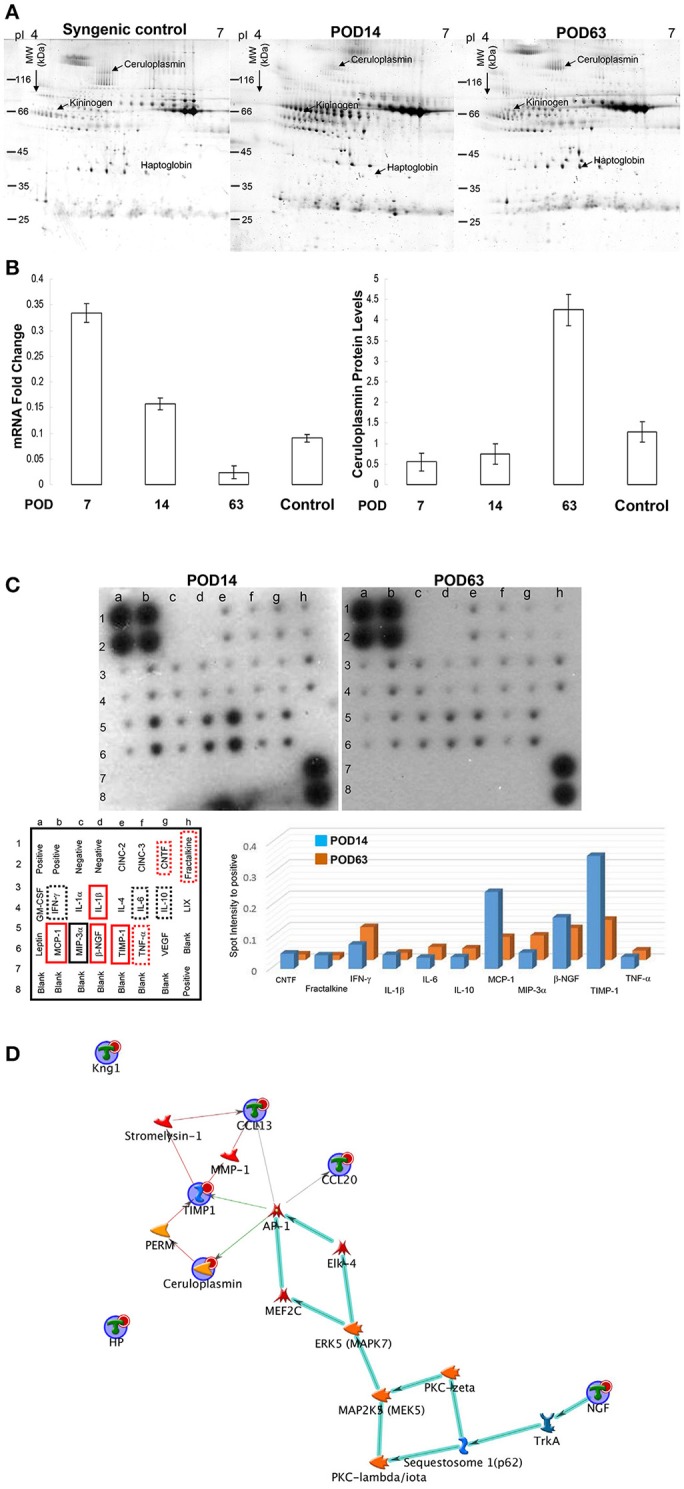
**(A)** Typical 2-DE protein profiles of rat serum samples. The protein volume was determined, and their intensities were quantified by sliver-stained 2-DE (Nonlinear Progenesis software). **(B)** The mRNA and protein levels of ceruloplasmin were normalized and the results represent the mean ± SD of three independent experiments on POD7, 14, 63 and control sample (syngenic control). **(C)** Cytokine levels were assessed by protein array on POD14 and POD63. The internal positive and negative controls were compared among array exposures. Cytokine/chemokine levels were normalized with respect to positive controls on the array membrane. The red solid line rectangle indicating the significantly higher values of cytokines in the rejection group compared to the tolerance sample whereas the black solid line rectangle showed significantly reduced level of cytokines on POD14 compared to that on POD63. The dotted line rectangles indicating the signals were slightly different with no statistical significance. The quantitative results indicating the different values were demonstrated as a bar chart. **(D)** Network analyses of differentially expressed proteins were applied with MetaCore software. Nodes represent proteins and lines between the nodes indicate direct protein–protein interactions. The various proteins on this map are indicated by different symbols demonstrating the functional class of the proteins. The biologic pathways in this network are mainly involved in immune regulation.

2-DE profiles were used to elucidate the particular serum protein involved in organ injury and to analyze the molecular mechanisms after OLT. Figure 2A represented a set of typical silver-stained gels from reproducible gel patterns of three independent experiments. More than 450 protein spots were shown in each gel. Of note, ceruloplasmin and haptoglobin, which have multiple physiological functions, were highly expressed in the tolerant samples whereas they were rarely identified in the rejection phase. In addition, we detected significant downregulation in the levels of kininogen for the POD63 sample compared to the POD14 subject. Moderate expression in the level of ceruloplasmin, haptoglobin and kininogen was identified in the syngenic control samples. Database searches with the peptide masses successfully identified the proteins with significant changes in protein volume. Since ceruloplasmin might play a critical role in determining oxidation regulation and immune responses, a Northern blot was conducted to determine whether regulation of ceruloplasmin protein correlated with simultaneous mRNA production. Our findings indicated that the expression of ceruloplasmin peaked on POD 7 and gradually decreased from days 7–63 following OLT. The possibility that this regulatory cycle might warrant the subsequent overexpression of ceruloplasmin on POD 63 was further confirmed by quantification of ceruloplasmin protein levels at different time points (Figure 2B).

### Overall cytokine/chemokine profile changes after OLT

Activation of cytokine and chemokine cascades would cause inflammatory responses and subsequent organ damage. To delineate the global changes in cytokines and growth factors, cytokine antibody array was applied to evaluate the serum concentrations of key inflammatory mediators after OLT. Several cytokines/chemokines, including the monocyte chemoattractant protein 1 (MCP-1), β-nerve growth factor (β-NGF), tissue inhibitor of metalloproteinases (TIMP-1), tumor necrosis factor (TNF-α), Ciliary neurotrophic factor (CNTF), fractalkine and Interleukin 1 (IL-1β), were obviously induced whereas MIP-3α was remarkably downregulated during the rejection period (POD14) compared with the tolerant group (POD63) (Figure 2C).

### Network analysis indicated that ceruloplasmin is critical in the OLT outcome

A list of the identified proteins aligned with the MASCOT and the significant changes in cytokines were indicated in Table 1. To further elucidate the relationship of differentially expressed proteins as well as the cytokines and their significance in the mechanisms associated with OLT outcomes, the targets were further analyzed using the MetaCore™ analytical tool. The algorithm builds biological networks from uploaded proteins/molecules and assigns a biological process to each network. As shown in Figure 2D, the network was generated utilizing the shortest path algorithm to map interactions among root proteins identified in the experiment. Protein-protein interaction networks demonstrated that ceruloplasmin seems to be a key protein mediating immune responses after OLT.

**Table 1:** Lists of proteins/cytokine expressed differentially between POD14 and POD63.

**Protein name**	**Accession number**	**Mw (kDa)**	**Fold change^a^**	**Biological function**
Ceruloplasmin	P13635	123.67	9.42 ± 0.56	It has ferroxidase activity oxidizing Fe^2+^ to Fe^3+^ without releasing radical oxygen species. May also play a role in fetal lung development or pulmonary antioxidant defense.
T-Kininogen I	P01048	47.76	−2.78 ± 0.32	They are inhibitor of thiol proteases.
Haptoglobin	P06866	38.56	6.56 ± 0.48	Controlling the equilibrium between tolerance and immunity to non-self antigens.
MCP-1 (CCL2)	P14844	11.03	3.44 ± 0.84	Chemotactic factor that attracts monocytes, but not neutrophils.
MIP-3α (CCL20)	P97884	8.0	−1.34 ± 0.15	Involved in the recruitment of both the proinflammatory IL17 producing helper T-cells (Th17) and the regulatory T-cells (Treg) to sites of inflammation. Required for optimal migration of thymic natural regulatory T cells (nTregs) and DN1 early thymocyte progenitor cells.
TIMP	P30120	23	5.17 ± 0.64	As a growth factor that regulates cell differentiation, migration and cell death and activates cellular signaling cascades via CD63 and ITGB1.
β-NGF	P25427	26	1.72 ± 0.39	A critical role in the regulation of both innate and acquired immunity. Inhibits metalloproteinase dependent proteolysis of platelet glycoprotein VI.

a Fold change indicated alterations of protein volume POD63 vs. POD14, respectively. Differences were considered significant at *p < 0.05.

### Survey of the oxidative stress via protein carbonylation

**Figure 3:**
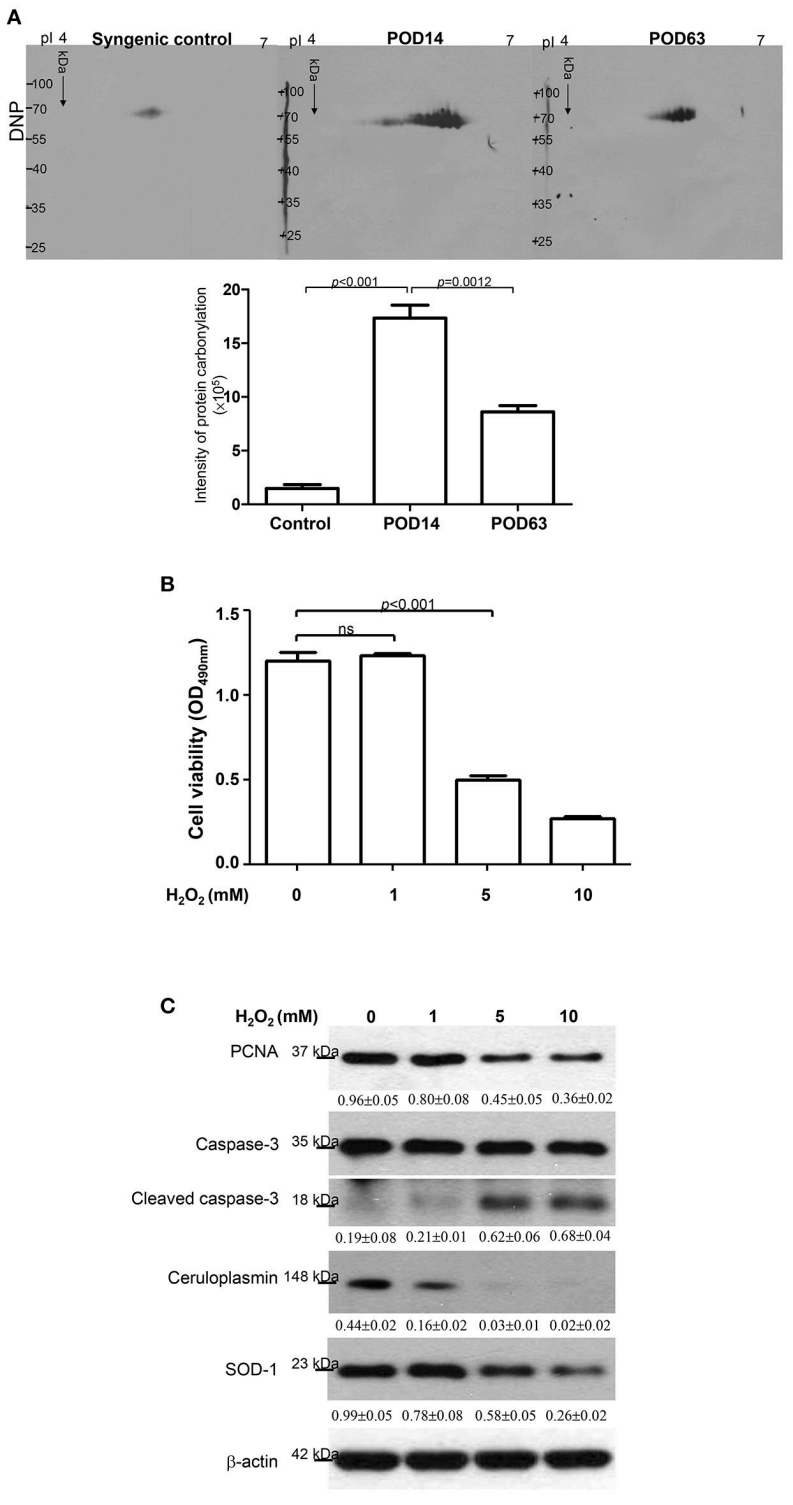
**(A)** Images of the 2-DE oxyblot. Analysis of protein oxidation levels in DNP-derivatized proteins among syngenic control, POD14 and POD63 samples. Obvious reduction in the carbonylation levels of proteins are observed in the tolerance group compared to rejection group. The quantitative results indicating the differentially carbonylated level of protein on syngenic control, POD14 and POD63 were demonstrated as the bar chart. **(B)** HepG_2_ cell viability was determined under the treatments of 0, 1, 5 and 10 mM H_2_O_2_. The result was quantified and indicated by the bar chart. **(C)** Western blotting analysis for PCNA, caspase-3, ceruloplasmin and SOD-1 levels under treatment of different concentrations of H_2_O_2_. The relative expression ratio to β-actin is shown at the bottom.

Previous reports have shown that various cytokines and chemokines would result in the cytotoxic effect of reactive oxygen species. This effect then induces leukocyte recruitment via the TNF-α and IL-1 signaling pathways and facilitates hepatic cell apoptosis (27, 33). The antioxidant activity of ceruloplasmin, which functions in concert with either scavengers of ROS or sequesters of free radicals, should correlate with the time point when the rejection was induced or overcome after OLT. Moreover, carbonylation of proteins due to oxidative modification would impair protein function in various metabolic processes, leading to organ failure after OLT. Changes in oxidized proteins can be detected by 2-DE oxyblots. As shown in Figure 3A, the degree of oxidation in proteins was quite low in the syngenic control group while the view of oxyblot obtained from the rejection group demonstrates that the specific protein oxidation was significantly upregulated. Interestingly, there was a remarkably decreasing tendency toward protein carbonylation in the tolerance group.

### Functional roles of ceruloplasmin in attenuating oxidative damage to hepatic cells

**Figure 4:**
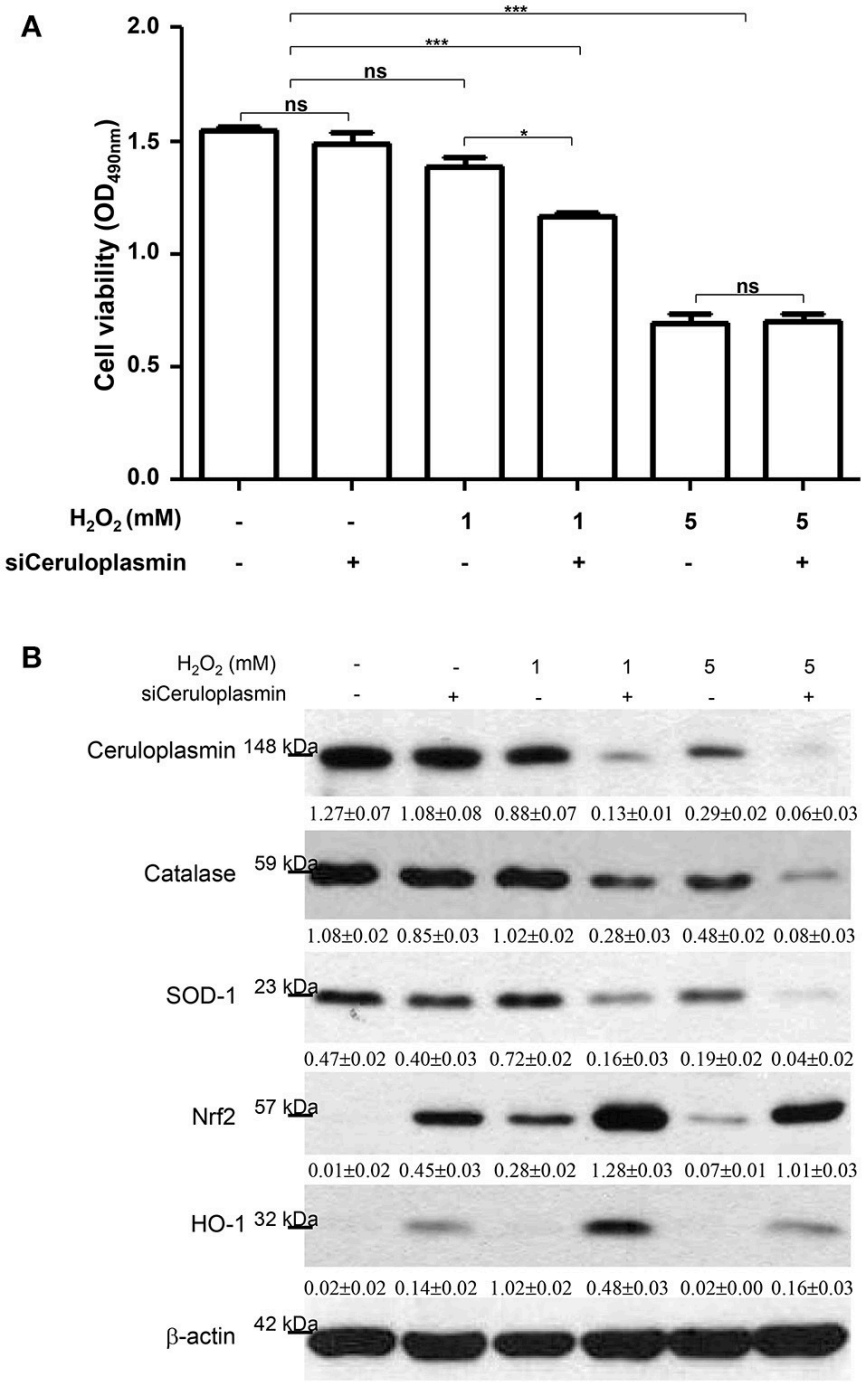
**(A)** HepG_2_ cell viability was determined under the treatments of 0, 1, and 5 mM H_2_O_2_ with or without ceruloplasmin siRNA (siCeruloplasmin) application. Results represent the mean ± SD of three independent experiments (*p < 0.05, ***p < 0.001). **(B)** The regulation of antioxidant enzymes and associated molecules under the administration of 0, 1, and 5 mM H_2_O_2_ with or without ceruloplasmin siRNA. β-actin was applied as the loading control. The relative expression ratio to β-actin is shown at the bottom.

According to the aforementioned findings, induced ceruloplasmin might effectively protect hepatic cells from oxidative insult and mediate tandem tolerance. We then further addressed this phenomenon in the HepG_2_ cell line, which exhibits biological characteristics and protein patterns similar to normal hepatocytes. HepG_2_ cells were then exposed to various concentrations of H_2_O_2_ (0, 1, 5, 10 mM) for 24 h. Cell viability was inhibited in a dose-dependent manner (Figure 3B). Meanwhile, Western blotting was performed to evaluate the functional impact of ceruloplasmin on HepG_2_ cell growth under strong oxidative stress. The results indicated that H_2_O_2_ obviously reduced the expression of ceruloplasmin compared to the control and activated the caspase-3 cascades leading to cell apoptosis. Superoxide dismutase-1 (SOD-1) was also exhausted in higher concentrations of H_2_O_2_ (Figure 3C). Taken together, a low level of ceruloplasmin could not protect the hepatic cells from damage caused by excessive oxidation. To further reveal the molecular mechanism associated with ROS-mediated cell injury, Ceruloplasmin siRNA was applied to verify the functional impact on HepG_2_ cell signaling pathways under H_2_O_2_ exposure. Knockdown of ceruloplasmin by small interfering RNAs significantly diminished cell survival compared to the cells without silent ceruloplasmin in response to a moderate concentration of H_2_O_2_ while no marked impact on cell viability was found in the ceruloplasmin abrogation group with respect to the control sample under exposure to a high concentration of H_2_O_2_ (Figure 4A). Again, Western blot analysis was performed using antibodies against several signaling proteins to detect ROS modulation. Remarkably decreased catalase and SOD-1 and obviously increased HO-1 as well as Nrf2 in the H_2_O_2_/ceruloplasmin siRNA–treated group compared to the only H_2_O_2_-applied group were detected, indicating that ceruloplasmin could effectively scavenge ROS via regulating the antioxidant genes and in turn sequester oxidative damage to hepatocytes (Figure 4B).

## Discussion

Liver allograft transplantation is an ultimate method for treating patients with end-stage liver diseases; nevertheless, nonspecific immunosuppressive drugs elicit severe complications such as increased risk of infection and tumorgenesis (2, 34, 35). Therefore, a major issue in transplantation is inducing and promoting specific immune tolerance while most tries could not successfully been implied into the clinic. Current reports have indicated that specific proteins play pivotal roles in complex immunological processes, such as the gain of self-tolerance, especially in the innate and adaptive immune response (36–38). We have made efforts to set up the functional proteome for the certification of serum proteins that might be responsible for spontaneous tolerance of OLT in rats (39).

The mechanism of acute liver damage after liver transplantation is thought to be involved in immediate cellular damage including necrosis, apoptosis and subsequent inflammatory infiltration (40). Histological examination revealed a serious rejection reaction manifested by dense cellular infiltration and severe apoptosis of hepatocytes within 2 weeks after OLT. Conversely, spontaneous tolerance occurred, resulting in remarkable diminution of inflammatory cytokine (Figure 2C) and hepatic cell apoptosis on POD63 (Figure 1).

Inflammatory cytokines have been shown to initiate the generation of ROS and result in an imbalance of the oxidative status (41, 42). Numerous documents have demonstrated that ROS could trigger and modulate graft failure following OLT (43). Our results also indicated that high concentrations of H_2_O_2_ would impact the hepatic cell growth. In the current study, a global cytokine assay demonstrated that inflammatory markers, including MCP-1, TNF-α, and IL-1β, were elevated while increased levels of carbonyl-modification of protein were found during the rejection period with respect to spontaneous tolerance, implying that inflammation-caused oxidative injury would lead to hepatic damage after OLT. These results are also in line with previous reports demonstrating that MCP-1 is highly expressed in the rejection of cardiac grafts (44). Therefore, therapeutic approaches aimed at attenuating oxidative stress in transplanted organs will be rational strategies for reducing the complications associated with OLT.

Given that serum from an OLT-tolerant rat could preclude an immune attack, we suggested that the specific serum proteins may contribute to the immune-regulatory function (11, 45). Therefore, 2-DE analysis combined with mass spectrometry was used to effectively investigate the potential protein factors and associated pathways leading to acute rejection or spontaneous tolerance after OLT. As expected, significant alterations of some acute-phase proteins related to immune responses, including ceruloplasmin, haptoglobin, and T-kininogen, were identified. To precisely investigate the molecular mechanisms of the immune response and regulation under OLT, MetaCore™ software that generates systematic profiling data was used. In Figure 2D, the relative enrichment with the uploaded proteome and cytokine data was mainly responsible for the interaction between ceruloplasmin and inflammation-associated molecules such as MAPK and AP-1. Based on the previous study, IL-1β could induce ceruloplasmin synthesis in the hepatic cells through several pathways including ERK1/2-, JNK1- and partially p38 MAPK-dependent activation of AP-1 (46–48). In this regard, upregulation of IL-1β on POD14 would contribute to the increase of ceruloplasmin, which results in drug-free tolerance on POD63. Previous studies have demonstrated that inhibition of proinflammatory cytokines could prolong the graft survival (49). Again, AP-1 is also critical in the CCL20 (MIP-3α) cascade that plays a role in the amplification of the immune response during renal allograft rejection (50). Regulation of ceruloplasmin by inflammation-caused redox stress may explain our findings of increased ceruloplasmin expression in tolerant grafts, where the intracellular copper level should be higher in a redox-compromised environment (51). Therefore, we suggest that AP-1–mediated induction of ceruloplasmin might contribute to the OLT tolerance.

IFN-gamma is a potent inducer of ceruloplasmin synthesis by monocytic cells and monocytic cell-derived ceruloplasmin may contribute to defense responses via its ferroxidase activity, which may drive iron homeostasis (52). Previous studies have indicated that iron signature is critical for the maintenance of operational tolerance (12, 53). Thus, the levels of proteins associated with iron homeostasis such as ceruloplasmin might predict the development of the tolerant state in the OLT model.

Ceruloplasmin is a blue copper-containing α2-glycoprotein mainly synthesized by the liver although its precise physiological functions remain unknown. Several of its activities, such as copper transport, immunosuppression and oxidation of organic materials, have been previously mentioned (54–56). In particular, an antioxidant capacity has been suggested as a critical function of ceruloplasmin to suppress the formation of free radicals due to its ferroxidase activity (57). Ceruloplasmin could effectively inhibit the ferrous ion-dependent formation of hydroxyl radicals in the Fenton reaction (58). Accordingly, the critical role of ceruloplasmin as a stoichiometric scavenger of superoxide radicals might be an important factor by which the rats could overcome the immune attack and the hepatocyte were no longer being damaged after OLT since these redox-sensitive cascades ultimately affect the outcome in the DA to PVG model (59). In contrast, both mRNA and protein level of ceruloplasmin on POD 7-14 in the DA to Lewis combination (rejection model) was much lower than that in the DA to PVG or the DA to DA group (Supplement Figure 1), suggesting that ceruloplasmin plays a pivotal role in the balance of redox status and tolerance induction. On the other hand, the discrepancy in peak period between mRNA level and protein expression of ceruloplasmin might be due to the great exhaust by ROS generated during rejection period (POD7~14). Moreover, post-translational modification which is important to immune regulation is also a possible reason that delays the peak in protein expression. In agreement with this notion, we observed that IL-6 was increased on POD63 and previous report has shown that IL-6 could promote protein fucosylation which contribute to various immune functions (14). In addition, protein carbonylation, a measure of protein modification by ROS, was markedly elevated at POD14, suggesting that ceruloplasmin is implicated in the antioxidant defense system which prevents damage to the transplanted liver by removing oxygen radicals.

As shown in our data, antioxidant enzymes such as catalase and SOD were significantly eliminated under administration of H_2_O_2_ and the silence of ceruloplasmin. In addition to the primary defense against ROS by antioxidant enzymes, some antioxidant reagents, including Nrf-2 and HO-1, were also evaluated in the H_2_O_2_/ceruloplasmin siRNA–treated samples with respect to the only H_2_O_2_-applied group. Activation of Nrf-2 cascade is an adaptative cellular response to oxidative injury and serves to maintain intracellular redox homeostasis (60). Given that ceruloplasmin knockout would cause the increase of ROS and trigger antioxidant cascades such as Nrf-2 and HO-1, it is likely that the antioxidant properties of ceruloplasmin make it a powerful hepatoprotective agent after OLT.

**Figure 5:**
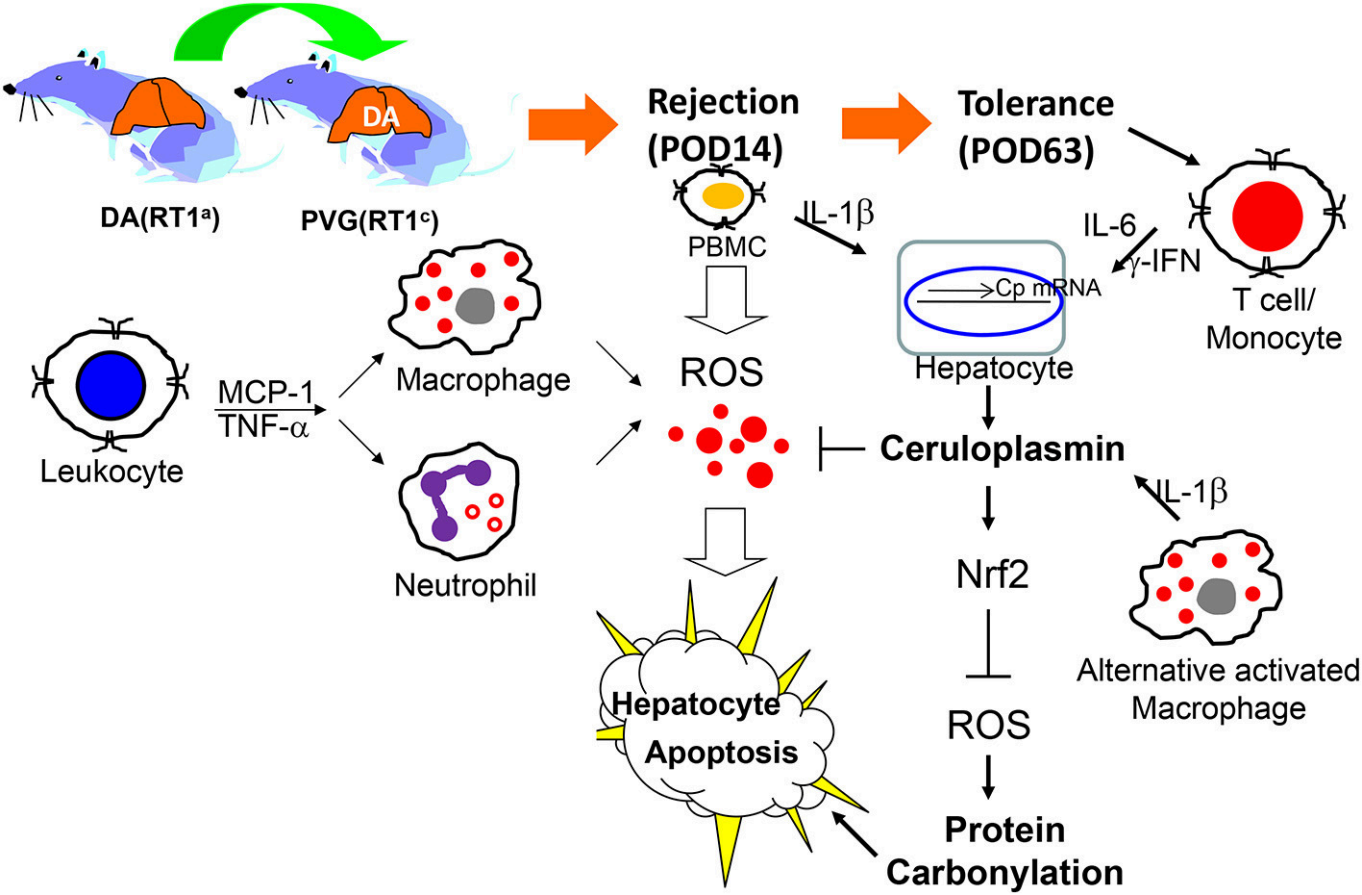
Schematic diagram of activated immune cell-mediated liver damage after OLT and ceruloplasmin could effectively alleviate the liver rejection via modulating the redox imbalance.

In conclusion, proteomics provided a logical analysis and revealed differentially expressed protein profiles related to OLT tolerance or rejection. Ceruloplasmin secreted by hepatocytes could effectively amend oxidative injury caused by cytokine-activated leukocytes or neutrophils (Figure 5). Our finding is not only the first document of identification of ceruloplasmin as a key factor in induction of spontaneous tolerance after OLT but may also provide novel insight into the roles of ceruloplasmin in organ transplantation related to inflammation, oxidative biology and immune regulation.

## Author contributions

T-LP and P-WW designed the protocol, helped conduct the experiment and prepared the manuscript, SG and C-LC provide supervision for the liver transplantation technique, T-HW and M-HC helped to conduct the statistical analysis, and T-LP was in charge of the whole experiment conduction and proofreading of the manuscript.

### Conflict of interest statement

The authors declare that the research was conducted in the absence of any commercial or financial relationships that could be construed as a potential conflict of interest.

## Supplementary material

Supplement Figure 1 The protein levels of ceruloplasmin among various combinations: DA to Lewis strains (DA → LEW), DA to PVG strains (DA → PVG), and DA to DA strains (DA → DA), were evaluated by Western blotting analysis. Five rats for each group were applied.
