# Supplementary figures and images for: Integrated Proteome and Cytokine Profiles Reveal Ceruloplasmin Eliciting Liver Allograft Tolerance via Antioxidant Cascades
Source: Front Immunol. 2018 Sep 26;9:2216. doi: 10.3389/fimmu.2018.02216 (PMC6168655; doi:10.3389/fimmu.2018.02216)

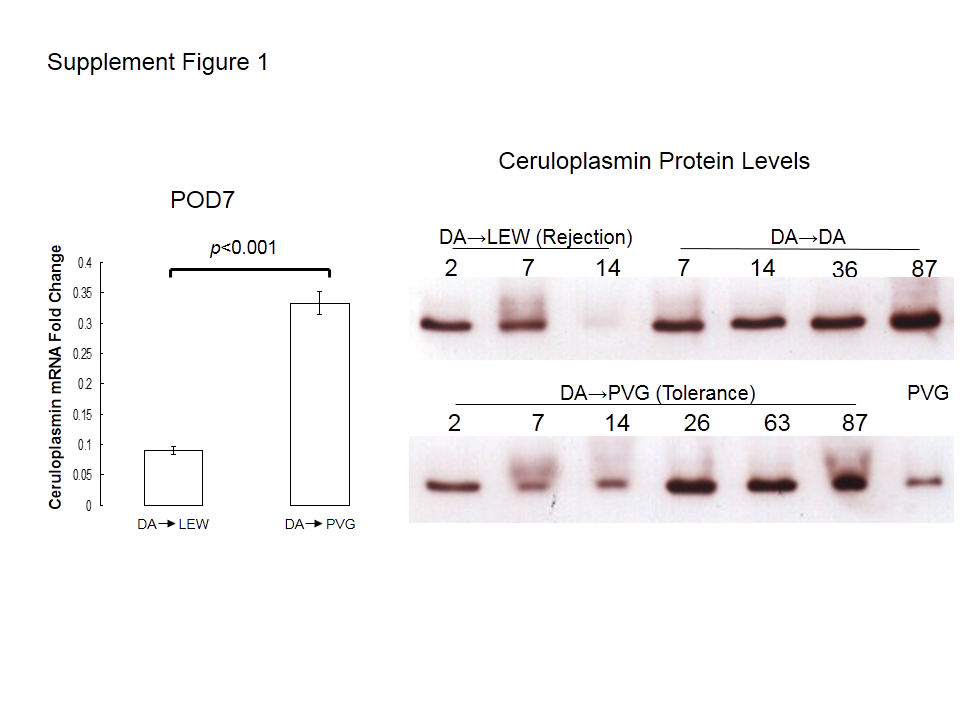

Supplement: Supplement Figure 1 — The protein levels of ceruloplasmin among various combinations: DA to Lewis strains (DA → LEW), DA to PVG strains (DA → PVG), and DA to DA strains (DA → DA), were evaluated by Western blotting analysis. Five rats for each group were applied. [file Image_1.TIF]
